# Dimensionless fluctuations balance applied to statistics and quantum physics

**DOI:** 10.1038/s41598-024-73790-1

**Published:** 2024-10-10

**Authors:** Marceliano Oliveira, George Valadares, Francisco Rodrigues, Márcio Freire

**Affiliations:** 1 UEA, Parintins, Amazonas Brazil; 2 UFAC, Rio Branco, Acre Brazil; 3 UVA, Sobral, Ceará Brazil; 4 UFC, Crateús, Ceará Brazil

**Keywords:** PDEs, Distributions, Boltzmann, Planck, Fermi–Dirac, Bose–Einstein, Schrödinger equation, Theoretical physics, Statistical physics

## Abstract

This work presents a new method called Dimensionless Fluctuation Balance (DFB), which makes it possible to obtain distributions as solutions of Partial Differential Equations (PDEs). In the first case study, DFB was applied to obtain the Boltzmann PDE, whose solution is a distribution for Boltzmann gas. Following, the Planck photon gas in the Radiation Law, Fermi–Dirac, and Bose–Einstein distributions were also verified as solutions to the Boltzmann PDE. The first case study demonstrates the importance of the Boltzmann PDE and the DFB method, both introduced in this paper. In the second case study, DFB is applied to thermal and entropy energies, naturally resulting in a PDE of Boltzmann’s entropy law. Finally, in the third case study, quantum effects were considered. So, when applying DFB with Heisenberg uncertainty relations, a Schrödinger case PDE for free particles and its solution were obtained. This allows for the determination of operators linked to Hamiltonian formalism, which is one way to obtain the Schrödinger equation. These results suggest a wide range of applications for this methodology, including Statistical Physics, Schrödinger’s Quantum Mechanics, Thin Films, New Materials Modeling, and Theoretical Physics.

## Introduction

In physics, often it is encountered situations where extending fundamental laws or developing new models that relate experimental observations in novel ways is needed^1^. Perturbation theory^2^, energy conservation^3^, Gauss divergence theorem^4^, Stokes’s curl theorem^5^, and Reynolds’s transport theorem^6^ are some examples of new models or different applications of the original idea. Another example can be seen in^7^. Each of these cases is better suited to a certain situation.

Observing some new materials applications^8^, it is clear that distributions play a central role in modeling this system^9^. Sometimes, some modification in the theoretical model due to experimental requirements is needed as a theoretical proposition^10^. This work emerges from the need to obtain distributions quickly.

This work presents a methodology that constructs a distribution function as a PDE solution using a generalized fluctuations balance analysis, as demonstrated in various case studies. This approach is applicable in a diverse range of scenarios due to the dimensionless nature of fluctuations that correlate two or more physical quantities.

Some case study observations contributed to the development of this work, like the Boltzmann distribution^11^ that was applied to Plank’s hypothesis, resulting in their Radiation Law^12^. Also in the case of ultraviolet catastrophe, as described in^13^, with $\left( E=kT\right)$ (where *k* is the Boltzmann constant), establishes a function of the pair of quantities energy and temperature $\left( E,T\right)$, leading to the form of Boltzmann distribution function $u\left( E,T\right) \propto e^{-E/kT}$^14^.

As the virtual work principle makes it possible to obtain Lagrange formalism for Classical Mechanics, the DFB method leads to PDEs which solutions are distributions.

### First concepts about dimensionless fluctuations

Some phenomena in nature can present similar aspects when compared with others. Observing when something is really equal between two different contexts or how one phenomenon can differ from the other is an important task in science and physics. Sometimes, verifying if there is some regime in which one phenomenon can be considered similar to another is also a goal.

**Fig. 1:**
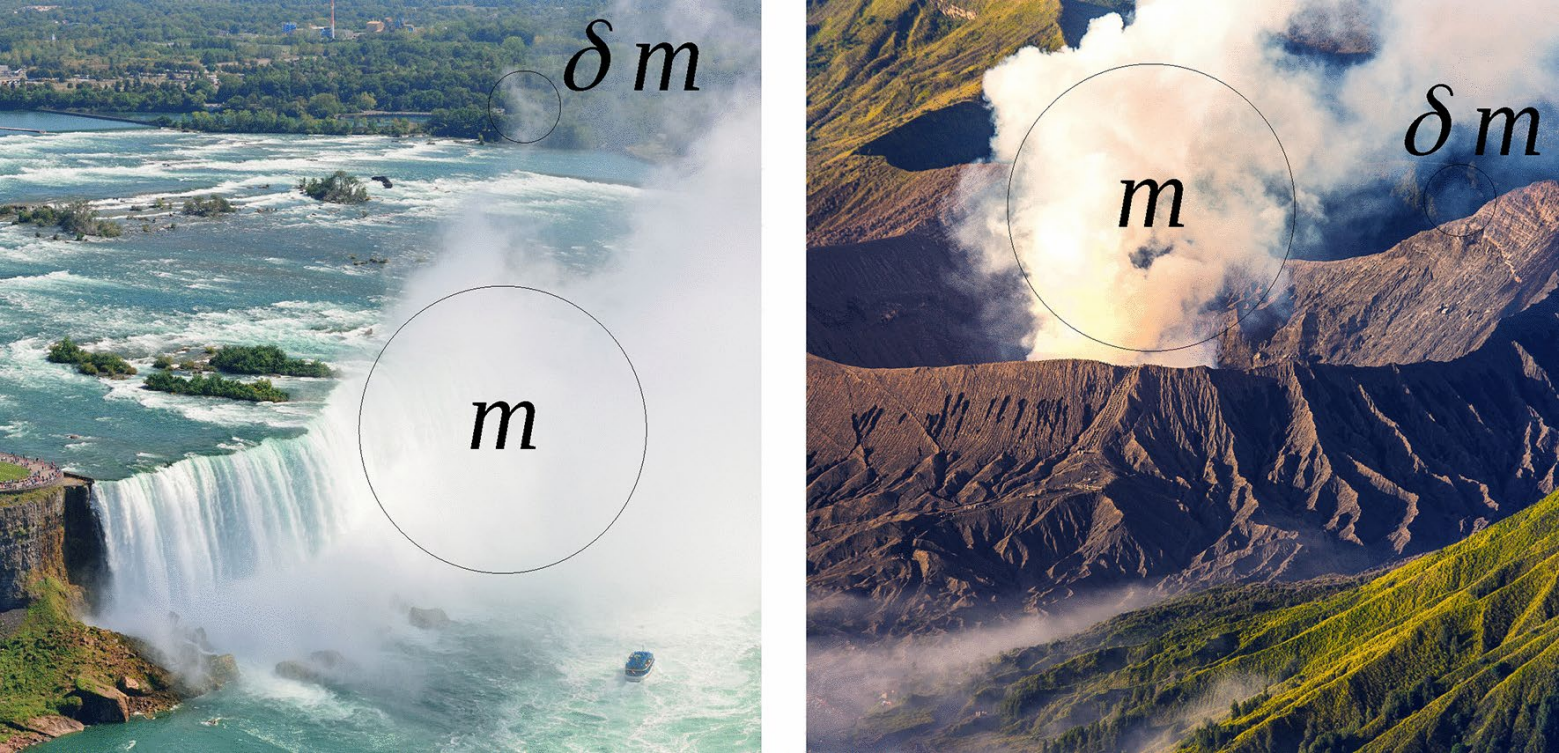
In the left figure, we can appreciate Niagara Falls, edited from freepik^15^, apparent spray configuration, similar to a cloud, and in the right figure, edited from freepik^16^, a volcano’s smoke is moving upward from inside. While both systems seem to display similar properties, a closer examination of their scales and other characteristics reveals that water frequently forms a large cluster of molecules known as a water drop, which is significantly larger than the clusters typically found in smoke constituents. In other words, we can expect that smoke is a better representation of gas than water spray. In both figures, *m* denotes a mass, and $\delta m$ is a very small portion of that mass.

When considering a system with many particles, like in Fig. 1, as a water steam or a smoke of materials, at first glance, these two cases remember a spray configuration, but looking precisely for the particle sizes, the velocity fields, and other aspects, it’s possible to distinguish one phenomenon from the other.

### Fluctuations balance

Continuing to observe cases with many particles, we can analyze a phenomenon called Dune Motion. In this case study, wind generates a current flux of sand, and after some time, a sand dune moves from a first local region to a second region in the neighborhood.

**Fig. 2:**
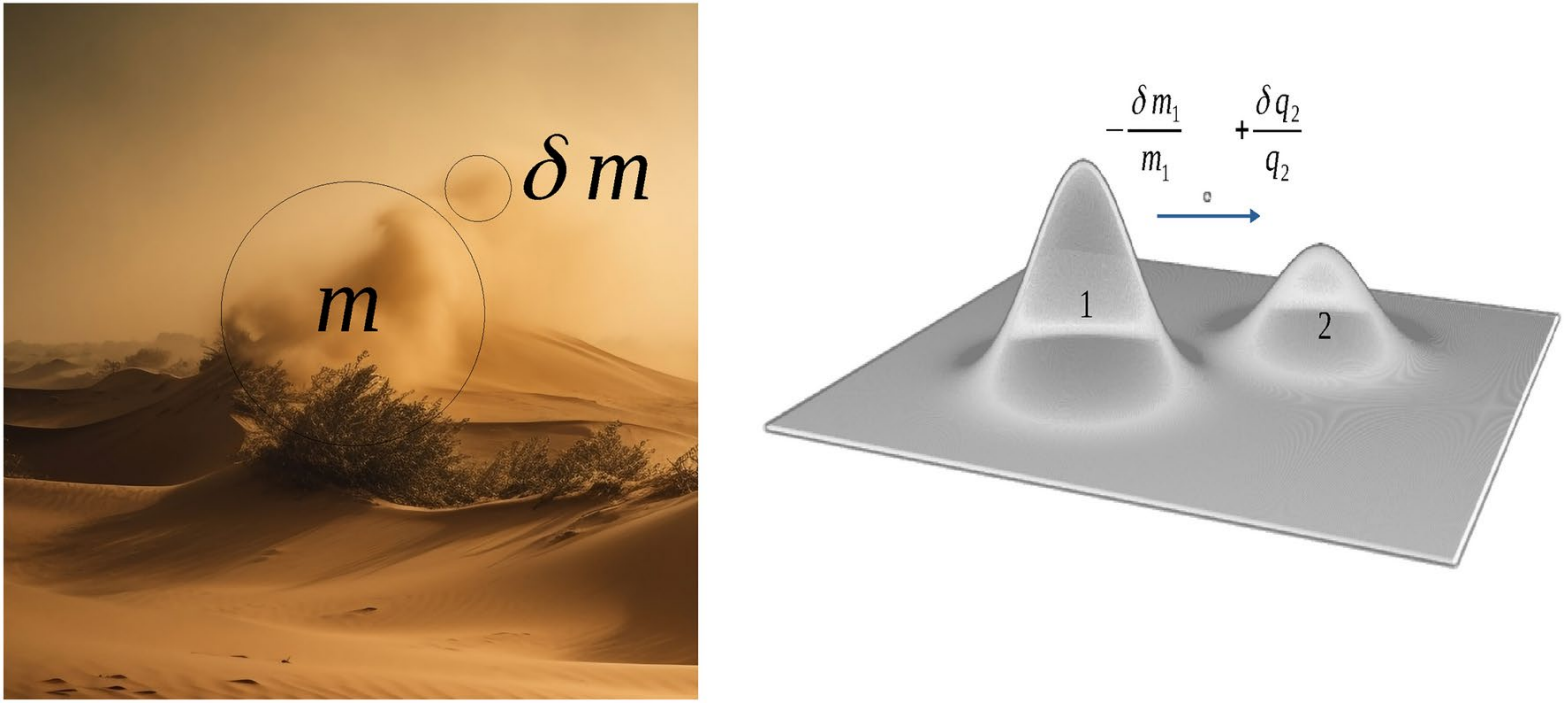
In the left we can see a dune in Sahara desert, edited from freepik^17^. Many articles or studies discuss the dune’s electrification process. When sand grains leave the region of the first dune, a mass fluctuation occurs. Entering a region of the second dune, electrification^18^ generates a charge fluctuation. During sand-dune transport, both mass and charge flux occur simultaneously. It is a typical case that illustrates a possible balance relating to two different physical quantities. In both figures, *m* denotes a mass and $\delta m$ a very small portion of that mass, while *q* denotes an electric charge and $\delta q$ a very small portion of that charge.

### Fluctuation mathematical aspects

As an example of property fluctuation, consider a particle that is significantly smaller than the original system. As shown in Fig. 2, when the diameter of a grain of sand is much smaller than the diameter of the dune that originated it, This generally applies to the following situations:

1 $$\begin{aligned} fluctuation = \frac{\delta \left( property\right) }{property}. \end{aligned}$$

Examples of properties include mass, charge, volume, density, and others. This form of presenting fluctuations is fundamentally dimensionless. When we consider the particles as pieces of a system, the normalization process naturally emerges. This is because, in the extreme case where the sum of all pieces, or a single piece, is as large as the system size, the maximum fluctuation value becomes the unit. So,

2 $$\begin{aligned} fluctuation = \sum _i^n \frac{\delta _i \left( property\right) }{property}=1. \end{aligned}$$

To apply the DFB method, two major conditions must be satisfied: (i) The proportionality factor must be adjusted when converting a proportionality into an equality. The DFB method requires the same adjustment as dimensional analysis, which requires tuning a constant factor. (ii) The balance must determine if the quantities under analysis increase or decrease. An increasing quantity must have a positive sign, and a decreasing quantity must have a negative sign, similar to constructing a differential equation. The DFB method strongly relates dimensionless factors to requirement (i), mirroring the nature of dimensional analysis^19^. Additionally, the DFB method depends on requirement (ii) because it involves relating fluctuations, similar to relating differentials. Both of these conditions are (i) necessary and (ii) sufficient to ensure the accuracy of the method.

## Boltzmann distribution PDE

Initially, by inspecting the first law of thermodynamics, we can verify their balance and understand how fluctuations emerge in this context.

### A closer view to first law of thermodynamics

As expressed by the First Law of Thermodynamics^20^, if a system gains a heat portion *dQ* at each cycle and realizes some work *dW* from the gained heat, the increase in the system’s internal energy due to the gained heat is given by,

3 $$\begin{aligned} dU=dQ-dW, \end{aligned}$$

or just,

4 $$\begin{aligned} dU=CdT-pdV. \end{aligned}$$

The *CdT* variable quantifies the heat gained due to the slight temperature fluctuations *dT* in certain material components of the system, which are associated with their thermal capacity *C*. The *pdV* term represents work realized by volume variation *dV* under pressure *p*.

Dividing Eq. (4) by *CT* reveals a small fluctuation of temperature, as can be seen in the form,

5 $$\begin{aligned} \frac{\delta T}{T}= \frac{\delta U}{CT} + \frac{p \delta V}{CT}. \end{aligned}$$

Equation (5) shows how temperature fluctuation is related to internal energy modification and work done by the system. The next step shows how fluctuations can contribute to understanding gas dynamics.

### Small fluctuation balance to gas system

As observed before, Eq. (5) relates three types of fluctuations: temperature fluctuation on the left hand side (LHS), internal energy fluctuation, and work fluctuation. To simplify, both terms in the RHS can be presented as a single composed term, designated as energy E.

At first glance, the energy-temperature fluctuations relation can be presented as follows,

6 $$\begin{aligned} \frac{\delta E}{E}\propto \frac{\delta T}{T}, \end{aligned}$$

however, in the specific case of an insulated gas system, a small expansion of the gas results in minimal positive work over its moving walls, pushing it outside of the center, similar to the concept of virtual work in classical mechanics. Also considering the physical facts, when ideal gas expands isolated in an adiabatic way, the temperature of the gas decreases, and this expansion costs internal energy a decrease too. A possible equation relating two states of internal energy $U_1, U_2$ where $U_2$ is internal energy after expansion and $U_1$ is initial internal energy,

7 $$\begin{aligned} U_2=U_1-\delta E, \end{aligned}$$

but for an ideal gas, internal energy only depends on temperature, so $U_2 \propto T_2$ and $U_1 \propto T_1$ lead to

8 $$\begin{aligned} T_2 \propto T_1 - \delta E, \end{aligned}$$

which is the same as,

9 $$\begin{aligned} \delta T \propto - \delta E. \end{aligned}$$

From (9), it is possible to see that a correct version of (6) is

10 $$\begin{aligned} \frac{\delta E}{E}\propto -\frac{\delta T}{T}. \end{aligned}$$

To transform proportionality from (10) into equality, one possibility is to multiply the temperature by the Boltzmann constant in both terms of the temperature fluctuation producing dimension of energy in terms of both sides of the equation. Then,

11 $$\begin{aligned} \frac{dE}{E}=-\frac{dkT}{kT}. \end{aligned}$$

The comparison between (9) and (6) and the choice of unity as the proportionality factor when going from (10) to (11) meet both the necessary and sufficient conditions set out at the end of the first section.

Where $\delta E, \delta T$ was considered so small that it can be approximated to *dE*, *dT*. As can be seen, a natural simplification of the constant *k* occurs. This is a strong aspect of fluctuation analysis because all fluctuation terms are dimensionless in essence. A new arrangement of terms separating differentials leads to

12 $$\begin{aligned} \frac{dE}{dT}=-\frac{E}{T}. \end{aligned}$$

Considering a distribution function such as $u\left( E,T\right)$ or other relevant dependencies of interest, a partial differential approach is most affordable for this purpose. Thus, (12) can change to,

13 $$\begin{aligned} \frac{\partial E}{\partial T}=-\frac{E}{T}. \end{aligned}$$

The chain rule, which aims to include the distribution function $u\left( E,T\right)$, is given by

14 $$\begin{aligned} \frac{\partial E}{\partial T}=\frac{\partial u}{\partial T}\frac{\partial E}{\partial u}. \end{aligned}$$

Replacing (14) in (13), the partial differential equation takes the form,

15 $$\begin{aligned} \frac{\partial u}{\partial T}=-\frac{E}{T}\frac{\partial u}{\partial E}. \end{aligned}$$

The Eq. (15) is the Boltzmann Distribution PDE, presented here for the first time. This is a new form of distribution study obtained from dimensionless fluctuation analysis. Now it is possible to find the Boltzmann distribution just by solving the partial differential equation (15).

A trial function solution can be employed based on two properties, as indicated by the earlier fluctuation analysis in Eq. (10). As an algebraic suggestion, we can utilize two arbitrary exponents, denoted as *a* and *b*, similar to the approach used in dimensional analysis. These exponents can be freely chosen at a later stage during the solution process, according to the expectations of the trial function solution method. Therefore,

16 $$\begin{aligned} u\equiv e^{\pm (E)^{a}\cdot (kT)^{b}}. \end{aligned}$$

Replacing (16) in (15),

17 $$\begin{aligned} \frac{\partial u}{\partial T}={\left\{ \begin{array}{ll}+k^{b}b T^{b-1}E^{a}u,\\ -k^{b}b T^{b-1} E^{a}u.\end{array}\right. } \end{aligned}$$

and,

18 $$\begin{aligned} \frac{\partial u}{\partial E}={\left\{ \begin{array}{ll}+k^{b}T^{b}a E^{a-1}u, \\ -k^{b} T^{b}a E^{a-1}u.\end{array}\right. } \end{aligned}$$

Equation (17) can be related to (18), just dividing both terms and simplifying in order to analyze the general solution. Then,

19 $$\begin{aligned} \frac{\partial u}{\partial T}=\frac{b}{a}\frac{E}{T}\frac{\partial u}{\partial E}, \end{aligned}$$

for both possibilities. ±.

A simple comparison between Eqs. (19) and (15) shows that the choice $b/a=-1$ turns Eq. (19) into the same form as (15). This choice is equivalent to $b=-a$, which simplifies the trial solution from Eq. (16) to,

20 $$\begin{aligned} u=e^{\pm \left( E/kT\right) ^{a}}. \end{aligned}$$

Both solutions, $u_+=e^{+\left( E/kT\right) ^{a}}$ and $u_-=e^{-\left( E/kT\right) ^{a}}$, are solutions to the Boltzmann Distribution PDE Eq. (15). Given temperature *T*, the negative solution is that it presents localization required to a distribution that is the same behavior as expected for a Gaussian function; the major surface value down the function curve is concentrated in a specific range of their independent variable. The simplest choice of power $a=1$ in a negative trial solution leads to Boltzmann’s distribution function.

21 $$\begin{aligned} u\propto e^{-E/kT}. \end{aligned}$$

The next step is to apply this Boltzmann distribution to the Planck hypothesis in order to derive Planck’s Radiation Law.

## Planck’s law as Boltzmann’s distribution PDE solution

Planck, when solving the ultraviolet catastrophe, explains two points. The first point pertains to their foundational achievements related to Boltzmann’s distribution. The second point is that he reached his formula heuristically by searching for a curve that satisfies experimental observations.

22 $$\begin{aligned} u\propto e^{+E/kT}. \end{aligned}$$

Replacing $E=h\nu$ in (22), the initial form of distribution became,

23 $$\begin{aligned} u=e^{h\nu /kT}. \end{aligned}$$

Planck solves the breaking region in the ultraviolet zone, which occurs because the *kT* term is much greater than $h\nu$, or just, $kT\gg h\nu$.

**Fig. 3:**
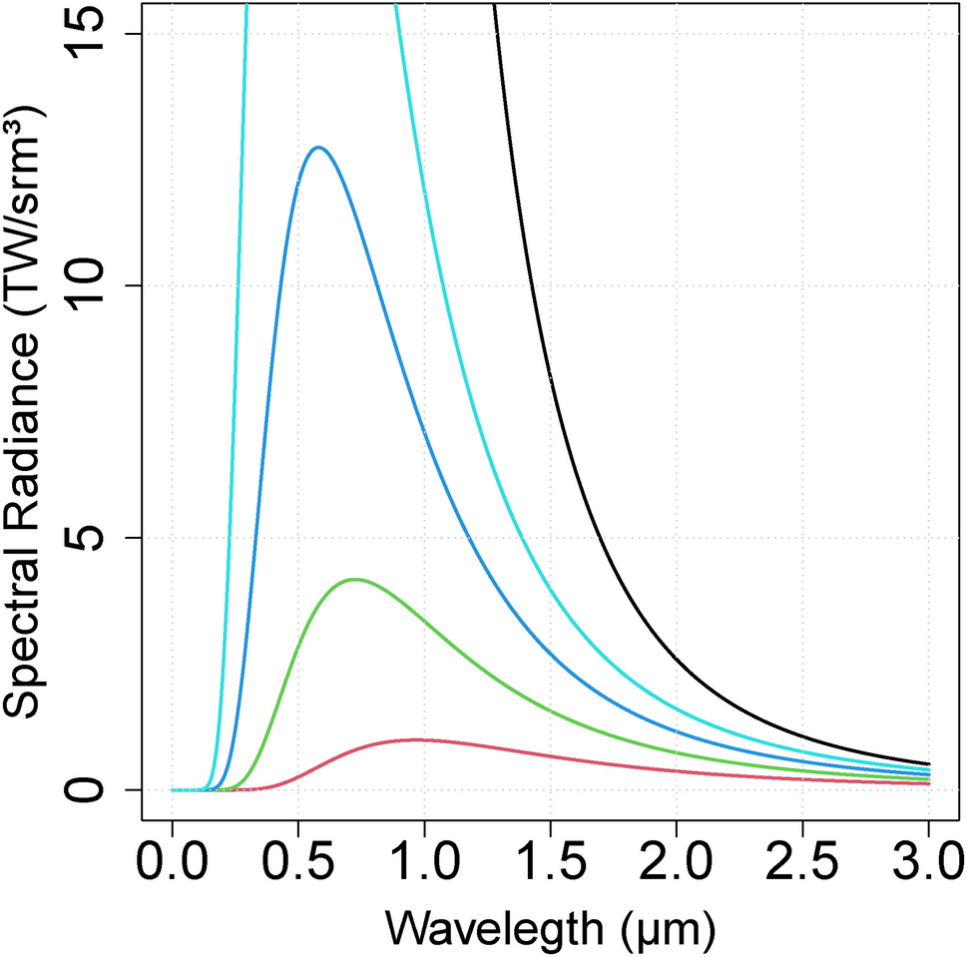
Classical radiation distribution, evidencing a temperature of 5000 K and a breaking point near the wavelength of 1 µm.

In the same fashion as Einstein explains easily mass-energy equivalence, expanding kinetic energy because of velocity *v* is much less than *c*^21^, let’s expand the Boltzmann distribution using the Planck hypothesis, with Taylor series at variable $\nu$ around the ultraviolet zone limit. So,

24 $$\begin{aligned} e^{h\nu /kT}=1+\frac{h\nu }{kT}+\frac{1}{2}\left( \frac{h\nu }{kT}\right) ^{2}+...+\sum _{n=3}^{\infty }\frac{1}{n!}\left( \frac{h\nu }{kT}\right) ^{n}. \end{aligned}$$

Considering $h\nu \ll kT$ (Fig. 3), let’s truncate terms to get an approximation for all terms with *n* equal two or higher, resulting in

25 $$\begin{aligned} e^{h\nu /kT}\simeq 1+\frac{h\nu }{kT}. \end{aligned}$$

Isolating the *kT* term,

26 $$\begin{aligned} kT\simeq \frac{h\nu }{e^{h\nu /kT}-1}, \end{aligned}$$

that is the thermal energy related to a photon in the gas at state $\mu$. Or just $E_{\mu }$,

27 $$\begin{aligned} E_{\mu }=\frac{h\nu }{e^{h\nu /kT_{\mu }}-1}. \end{aligned}$$

This equation represents Planck’s Law of Radiation, which provides the energy per photon state $\mu$ in the gas and is valid in the ultraviolet zone. Through his law, Planck establishes a mathematical relationship that explains a phenomenology not previously resolved by classical formulations.

To reinforce the importance of the Boltzmann PDE presented in (15), let us verify in the next steps that the Planck Radiation Law in Eq. (27) also a solution of the Boltzmann PDE.

When Planck started from Boltzmann distribution and obtained his radiation law, he defined the case study as a photon gas system that has compatibility with the Boltzmann gas model. From the perspective that Planck’s law originated from Boltzmann distribution, let’s investigate whether Planck’s law satisfies Boltzmann PDE Eq. (15),

28 $$\begin{aligned} \frac{\partial u}{\partial T}=-\frac{E_{\mu }}{T_{\mu }}\frac{\partial u}{\partial E}, \end{aligned}$$

Remembering Planck’s law from (27),

29 $$\begin{aligned} E_{\mu }\equiv u=\frac{h\nu }{e^{h\nu /kT_{\mu }}-1}. \end{aligned}$$

Replacing (29) in (28), gives

30 $$\begin{aligned} \frac{\partial u}{\partial T}=\frac{h^2\nu ^2}{kT_{\mu }^2}\frac{e^{h\nu /kT_{\mu }}}{\left( e^{h\nu /kT_{\mu }}-1\right) ^2}, \end{aligned}$$

and,

31 $$\begin{aligned} \frac{\partial u}{\partial E}=-\frac{h\nu }{kT_{\mu }}\frac{e^{h\nu /kT_{\mu }}}{\left( e^{h\nu /kT_{\mu }}-1\right) ^2}, \end{aligned}$$

Finally, relating (31) and (30) closes the question.

32 $$\begin{aligned} \frac{\partial u}{\partial T}=-\frac{h\nu }{T_{\mu }}\frac{\partial u}{\partial E}. \end{aligned}$$

Clearly, Planck’s radiation law satisfies the Boltzmann PDE equation with $h\nu$ as energy *E*.

## Fermi–Dirac distribution

The next case consists of verifying how Fermi–Dirac and Bose–Einstein are related to a similar format as that obtained in the equation of Planck’s law (27). The emphasis consists of using the Boltzmann PDE to inspect how close Fermi–Dirac and Bose–Einstein are to Plank’s law format. The central question is whether Fermi–Dirac and Bose–Einstein are also solutions of the Boltzmann PDE, or if they are just similar with a little modification in energy. Starting with the Fermi–Dirac distribution,

33 $$\begin{aligned} u=\frac{1}{e^{\left( \varepsilon -\mu \right) /kT}+1}. \end{aligned}$$

Differentiating it by temperature,

34 $$\begin{aligned} \frac{\partial u}{\partial T}=\frac{e^{\left( \varepsilon -\mu \right) /kT}}{\left( e^{\left( \varepsilon -\mu \right) /kT}+1\right) ^2}\frac{\left( \varepsilon -\mu \right) }{kT^2}, \end{aligned}$$

and energy,

35 $$\begin{aligned} \frac{\partial u}{\partial E}=-\frac{e^{\left( \varepsilon -\mu \right) /kT}}{\left( e^{\left( \varepsilon -\mu \right) /kT}+1\right) ^2}\frac{1}{kT}, \end{aligned}$$

and finally relating both,

36 $$\begin{aligned} \frac{\partial u}{\partial T}=-\frac{ \left( \varepsilon -\mu \right) }{T}\frac{\partial u}{\partial E}. \end{aligned}$$

As a result, it’s possible to observe that Fermi–Dirac Distribution satisfies the Boltzmann PDE.

## Bose–Einstein distribution

After success in verifying Fermi–Dirac Distribution as a solution of Boltzmann PDE, this case study consists of Bose–Einstein Distribution analysis for the same goal, which is to verify if this one is a solution of Boltzmann PDE. The Bose–Einstein distribution can be presented as follows,

37 $$\begin{aligned} u=\frac{1}{e^{E/kT}-1}, \end{aligned}$$

with energy *E* as $(\varepsilon -\mu )$. The differentiation of (37) in temperature gives,

38 $$\begin{aligned} \frac{\partial u}{\partial T}=\frac{e^{\left( \varepsilon -\mu \right) /kT}}{\left( e^{\left( \varepsilon -\mu \right) /kT}-1\right) ^2} \frac{\left( \varepsilon -\mu \right) }{kT^2}, \end{aligned}$$

and energy,

39 $$\begin{aligned} \frac{\partial u}{\partial E}=-\frac{e^{\left( \varepsilon -\mu \right) /kT}}{\left( e^{\left( \varepsilon -\mu \right) /kT}-1\right) ^2} \frac{1}{kT}, \end{aligned}$$

relating both (38) and (39),

40 $$\begin{aligned} \frac{\partial u}{\partial T}=-\frac{ \left( \varepsilon -\mu \right) }{T} \frac{\partial u}{\partial E}. \end{aligned}$$

Finally, it’s possible to observe that Bose–Einstein Distribution also satisfies the Boltzmann PDE.

Another question that is possible to investigate is related to modeling thermodynamic systems. Macroscopic variables like temperature, pressure, and volume can be measured by experimentation. Otherwise, it’s a well-known fact that these are manifestations of microstate configuration. As in the case of temperature, which is the average molecular kinetic energy.

Entropy is a quantity that relates microstates to macrostates^22^. It was possible to obtain the Boltzmann distribution as a solution to a PDE that originated from a dimensionless fluctuation balance. In the next session, let’s investigate how to obtain Boltzmann’s entropy law for the distribution of microstates emerging as solutions from the Boltzmann’s Entropy Distribution PDE, which is another new result presented here for the first time.

## Boltzmann’s entropy law

Previous sections were successful when verifying some known distributions and their close relationship with the Boltzmann PDE, which proves their high value in statistical physics. The goal now is to obtain Boltzmann’s entropy law, which starts with the concept of fluctuation balance and relates it to a distribution.

Assuming a certain gas volume and its expansion, an increase in volume leads to an increase in entropy because, as the volume expands, the number of possible states in the new configuration increases too. However, this increase in entropy, caused by volume expansion, consumes the thermal energy of the gas, causing it to decay. So, similarly to (10),

41 $$\begin{aligned} \frac{\delta \left( E_{S}\right) }{E_{S}}=-\frac{\delta \left( E_{T}\right) }{E_{T}}. \end{aligned}$$

Comparing it to fluctuations of Eq. (10), instead of energy in LHS, here is proposed energy related to entropy $E_S$, and in RHS, instead of temperature, it was adopted energy related to temperature decrease $E_T$. As thermal energy $E_T$ increases in volume, presenting more possible microstates, it is the same that says entropy energy $E_S$ increases.

The choice of the proportionality factor as unity and balance, as realized in Eq. (10) as referred to above, satisfies both the necessary and sufficient conditions for Eq. (41) as established in the first section.

At proper limit we can adjust this equation to,

42 $$\begin{aligned} \frac{dE_{S}}{E_{S}}=-\frac{dE_{T}}{E_{T}}. \end{aligned}$$

A distribution $\Omega$ will depend on the energy displaced to entropy when volume increases, $E_{S}$, and the thermal energy costs to volume increase, $E_{T}$. Given $\Omega \equiv \Omega \left( E_{S},E_{T}\right)$ the change from total to partial differential equation is most affordable for this purpose. So,

43 $$\begin{aligned} \frac{\partial E_{S}}{\partial E_{T}}=-\frac{E_{S}}{E_{T}}. \end{aligned}$$

The chain rule related to this case that introduces implicit dependencies of distribution $\Omega$ is,

44 $$\begin{aligned} \frac{\partial E_{S}}{\partial E_{T}}=\frac{\partial \Omega }{\partial E_{T}}\frac{\partial E_{S}}{\partial \Omega }. \end{aligned}$$

Replacing (44) in (43) we get a final form of partial differential equation presented here in a novel way as Boltzmann’s Entropy Distribution PDE,

45 $$\begin{aligned} \frac{\partial \Omega }{\partial E_{T}}=-\frac{E_{S}}{E_{T}}\frac{\partial \Omega }{\partial E_{S}}. \end{aligned}$$

Note that it differs from Boltzmann PDE in (15) observing that in this case the fraction in the RHS term relates energy of entropy and energy of temperature instead of energy and temperature in the previous case. The trial function method is the approach to solving this equation. So,

46 $$\begin{aligned} \Omega \left( E_{S},E_{T}\right) \equiv e^{E_{S}^{a}E_{T}^{b}}. \end{aligned}$$

To obtain the PDE solution using the trial function from (46), it is possible to set: first in terms of $E_{T}$ given $bE_{S}^{a}E_{T}^{b-1}\Omega$ and after in terms of $E_{S}$, to find $aE_{S}^{a-1}E_{T}^{b}\Omega$. After the trial function is used to relate both derivatives, it is possible to establish that

47 $$\begin{aligned} \frac{\partial \Omega }{\partial E_{T}}= \frac{b}{a}\frac{E_{S}}{E_{T}}\frac{\partial \Omega }{\partial E_{S}}. \end{aligned}$$

A simple comparison between (47) and (45) establishes that $b/a=-1$ is a necessary choice, which is the same as $b=-a$. So, finally, we obtain the solution of PDE in (45) in the format,

48 $$\begin{aligned} \Omega \left( E_{S},E_{T}\right) =e^{E_{S}^a/E_{T}^a}, \end{aligned}$$

with their simplest solution when the power coefficient *a* assumes unitary value. So,

49 $$\begin{aligned} \Omega \left( E_{S},E_{T}\right) =e^{E_{S}/E_{T}}. \end{aligned}$$

At the next step, the connection between this distribution and Boltzmann’s entropy law is possible. Using as first principles dimensional analysis and thermodynamics laws. From the entropy fundamental concept defined in thermodynamics, $dQ=TdS$, we can see that $\left[ dQ\right] =dE_{S}$ establishing the relation between heat energy and entropy energy, and as a macroscopic version, we can set the dimensional dependence of entropy energy as $E_{S}\propto \left[ T\right] ^{a}\left[ S\right] ^{b}$ which dimensional solution points to $E_{S}=TS$. Another requirement is to find thermal energy, $E_{T}$, which we obtain as $E_{T}=kT$. Given,

50 $$\begin{aligned} \Omega \left( E_{S},E_{T}\right) =e^{TS/kT}=e^{S/k}. \end{aligned}$$

To isolate entropy *S* in the final form, let we assume the inverse function from (50), resulting

51 $$\begin{aligned} S=k\ln \left| \Omega \right| . \end{aligned}$$

As a result, we recover Boltzmann’s Entropy Law, for first time directly obtained as PDE solution that emerges from the Dimensionless Fluctuation Balance method.

## Schrödinger’s formalism

This case study shows how to use dimensionless fluctuation balance with the uncertainty principle^23^ in quantum mechanics. The principle was derived from experiments and Gaussian width and height relationships and serves as a robust foundation^24^. In this scenario, DFB generates a PDE, and their solution leads to the Schrödinger formalism. So,

52 $$\begin{aligned} \delta x \delta p \ge \frac{\hbar }{2}, \end{aligned}$$

and

53 $$\begin{aligned} \delta E \delta t \ge \frac{\hbar }{2}. \end{aligned}$$

To analyze the relationship between position-momentum (52) and energy-time relation (53), an important consideration is to remember that the electrons that leave an oven and compose the beam gun only have kinetic energy because these ones are in unbounded states, so $E=K+0.$ As a consequence, $\delta E=\delta \left( mv^{2}/2 \right) = mv \delta v.$ Replacing it in (53) leads to $mv \delta v \delta t = \hbar /2$, and remembering that $\delta x = v \delta t$ and $\delta p = m \delta v$, recovering the other uncertainty relation $\delta x \delta p = \hbar /2$. The fact that particles in a beam are not in bounded states links both uncertainty relations.

Considering an oven containing confined electrons in an unbonded state, if one single electron leaves the oven, crossing an aperture in a beam direction, this electron gains linear momentum in the beam direction after crossing this aperture $+\delta p$. Otherwise, the system inside the oven, when losing this electron, loses an amount of internal energy $-\delta E$ because the electrons take away their kinetic energy from the oven to the outer region composing the beam. Considering that there are many electrons inside the oven before this electron scape, it is adequate to model this event, balancing their fluctuations. So,

54 $$\begin{aligned} \frac{\delta p}{p} \propto -\frac{\delta E}{E}. \end{aligned}$$

As next steps, we start replacing uncertainty relations from (52) and (53), in the limit that we equate both. So,

55 $$\begin{aligned} \delta E \delta t= \delta x \delta p. \end{aligned}$$

Multiplying (55) by the inverse of *E*, and after a little algebra, we get

56 $$\begin{aligned} \frac{\delta E}{E}= 2\frac{\delta p}{p}. \end{aligned}$$

After analyzing uncertainty relations, the Eq. (56) reveals the proportionality factor 2. The proportionality factor is a necessary condition (i); the sufficient conditions treat the balance mapping the energy displacement, as realized before in (54). So, from (54) that contain energy balance, it turns to (57) after proportionality factor 2 discovered from uncertainty relations analysis in (56).

57 $$\begin{aligned} \frac{\delta E}{E}= -2\frac{\delta p}{p}. \end{aligned}$$

After a little algebra, and considering the proper limit when $\delta E$ and $\delta p$ can be assumed as differentials, we get

58 $$\begin{aligned} \frac{dE}{dp}= -2\frac{E}{p}. \end{aligned}$$

Assuming that we have some distribution function, $\Psi \equiv \Psi \left( p, E \right)$, the partial derivatives turn (58) into a most interesting format,

59 $$\begin{aligned} \frac{\partial E}{\partial p}= -v. \end{aligned}$$

Assuming a chain rule using $\Psi$, relating *E* and *p* we get,

60 $$\begin{aligned} \frac{\partial E}{\partial p} = \frac{\partial \Psi }{\partial p}\frac{\partial E}{\partial \Psi }. \end{aligned}$$

Replacing (60) in (59), we finally get the Schrödinger Formalism PDE,

61 $$\begin{aligned} \frac{\partial \Psi }{\partial p}=-v\frac{\partial \Psi }{\partial E}. \end{aligned}$$

We can easily see that a simple solution for this one is given by,

62 $$\begin{aligned} \Psi =e^{ipx/\hbar }e^{-iEt/\hbar }. \end{aligned}$$

This distribution equation is very important because, from it, we can obtain all properties of the system, including Schrödinger Equation Formalism, which represents a free particle case. From the expected value as a statistical concept, $\left\langle p\right\rangle =\int _{-\infty }^{+\infty }p\Psi \left( x,t\right) dx$, we can see that distribution plays a central role. Because of the complex variable format at (62), we can extend this to square integration as $\left\langle p\right\rangle =\int _{-\infty }^{+\infty }\Psi ^{*}\left( x,t\right) {\hat{p}}\Psi \left( x,t\right) dx$. In the same fashion for $\left\langle H\right\rangle$ and $\left\langle V\right\rangle$, putting it all together,

63 $$\begin{aligned} \left\langle H\right\rangle = \left\langle p\right\rangle ^{2}/2m+\left\langle V\right\rangle , \end{aligned}$$

in a square integrable system,

64 $$\begin{aligned} \int _{-\infty }^{+\infty }\Psi ^{*}\left( x,t\right) {\hat{H}}\Psi \left( x,t\right) dx=\frac{1}{2m}\int _{-\infty }^{+\infty }\Psi ^{*}\left( x,t\right) {\hat{p}}^{2}\Psi \left( x,t\right) dx+\int _{-\infty }^{+\infty }\Psi ^{*}\left( x,t\right) {\hat{V}}\Psi \left( x,t\right) dx. \end{aligned}$$

By the concept, the ${\hat{p}}$ operator must extract eigenvalue from distribution, as in the form ${\hat{p}}^{2}\Psi \left( x,t\right) =p^{2}\Psi \left( x,t\right)$. Because of the format of $\Psi$ in (64), only a specific ${\hat{p}}$ operator can extract the *p* eigenvalue. So,

65 $$\begin{aligned} \Psi \left( x,t\right) =e^{ipx/\hbar }e^{-iEt/\hbar }\Leftrightarrow {\hat{p}}\equiv -i\hbar \frac{\partial }{\partial x}. \end{aligned}$$

In the same fashion, ${\hat{H}}$ operators extract energy *E* and ${\hat{V}}$ operators impose potential energy *V* for bounded state cases; replacing it in Eq. (64), the Schrödinger equation from expected values gives,

66 $$\begin{aligned} \int _{-\infty }^{+\infty }\Psi ^{*}\left( x,t\right) \left[ i\hbar \frac{\partial }{\partial t}+\frac{\hbar }{2m}^{2}\frac{\partial { ^2}}{\partial x{ ^2}}-V\left( x,t\right) \right] \Psi \left( x,t\right) dx=0, \end{aligned}$$

In equation (66), the time-dependent Schrödinger equation was obtained. This formalism can yield both dependent and independent Schrödinger equations. From (66), follows

67 $$\begin{aligned} i\hbar \frac{\partial }{\partial t}\Psi \left( x,t\right) =-\frac{\hbar }{2m}^{2}\frac{\partial { ^2}}{\partial x{ ^2}}\Psi \left( x,t\right) +V\left( x,t\right) \Psi \left( x,t\right) . \end{aligned}$$

As a result, the time-dependent Schrödinger Equation was obtained here from fluctuations and Heisenberg Uncertainty Relations, in a unique and novel way.

## General aspects of the DFB method

Compared to other methods, such as variational methods or perturbation theory, DFB establishes a formalism more quickly. This advantage occurs because both perturbation and variational methods connect changes in many variables using calculus in the case of perturbation and a path integral that fixes the extreme points in the case of variational methods.

Perturbation methods are valid when there are slight changes in a single variable that are close to a known solution. In the variational case, the method’s validity is still dependent on both fixing the path integral’s extreme points and going through an extra extremization step to get to the formalism.

The DFB method, a dimensionless fluctuation method, incorporates extremization into a very small fluctuation concept and fulfills its requirements through balance. It is a method with strong conservation and dimension analysis aspects that relate directly to the variables of interest without requiring another advanced calculus procedure.

Small fluctuations, which ensure conservation and lead to a valid PDE formalism that remains valid, are the only constraints on DFB’s validity. Here, we present the energy square analysis of fluctuation in Hamiltonian formalism to demonstrate how conservation is solely dependent on the small fluctuation requirement,

68 $$\begin{aligned} H \left[ \frac{\delta H^2}{H^2} - \frac{\delta H}{H} \right] = \delta K + \delta U. \end{aligned}$$

To obtain (68), we just multiply a Hamiltonian by *H*, then take a small delta on both sides and divide it by $H^2$, so after a little algebra, it’s possible to reach (68). Analyzing (68), it is easy to see that if *H* fluctuation is small, the left-hand side term is null because, naturally, $H^2$ fluctuation converges to zero faster than *H* fluctuation. So, the equation $\delta K = - \delta U$ shows that every kinetic energy increase costs a potential energy decrease. In other words, small fluctuations in the Hamiltonian ensure energy conservation.

The DFB method’s connection to the PDEs is another important aspect. To simulate traditional distribution relations based on integrals, the Monte Carlo method is usually used. On the other hand, PDE models allow for discretization using the finite difference method or another method, reducing the computational cost of simulation.

## Concluding remarks

After our tests applying the DFB method, the results were verified with success by the principal of physics distributions, finding a collection of procedures registered in this paper. At least this work shows a new way to relate two quantities using dimensionless numbers that enable us to obtain distributions as differential equation solutions. This is not only a practical tool; it also allows us to understand some meaning beyond distributions. As an example, we can see that when some particle, energy, or other system entity was confined in some regions, like localized phenomena, or when required to interact with some other entities not in large space fields, distributions emerged as the natural imposition of a space region on a group of entities, every time the system size was as small as needed to impose statistical reality on every entity within it.

## Data Availability

All data generated or analyzed during this study are included in this published article.
